# The Direct Tendon Suture and Paratenon Repair Technique for Acute Tendinous Mallet Finger: A Case Series

**DOI:** 10.3390/jcm13113215

**Published:** 2024-05-30

**Authors:** Seungjun Lee, Seokchan Eun

**Affiliations:** Department of Plastic and Reconstructive Surgery, Seoul National University College of Medicine, Seoul National University Bundang Hospital, Seongnam 13620, Republic of Korea; winters88@naver.com

**Keywords:** tendon injuries, mallet finger, suture techniques

## Abstract

(1) **Introduction:** Tendinous mallet finger is a frequent deformity that occurs after an extensor tendon injury during sports or daily life activities. Despite the existence of numerous non-operative and operative techniques to address this deformity, there is a controversy on its optimal management. In this study, we aimed to present a direct tendon suture technique using the distal interphalangeal (DIP) joint open approach for treating tendinous mallet finger injury. (2) **Methods**: Between 2019 and 2021, 19 patients with closed non-fracture tendinous mallet fingers underwent the direct tendon and paratenon repair technique. After skin incision, we opened the paratenon with lazy S shape incision and found the ruptured proximal and distal tendon ends. We reapproximated the tendons using a simple interrupted suture with Prolene #6/0. After that, we meticulously performed paratenon repair using PDS #6/0 for preventing readherence. Temporary trans-articular Kirschner wire fixation was used for 4 weeks. (3) **Results**: All patients were followed-up for 3–8 months (mean: 4.8 months). The mean final extension lag was 6.5 degrees, and the overall rate of cases with excellent and good outcomes using Crawford’s criteria was 85%. (4) **Conclusions**: In conclusion, this surgical approach could be a reliable alternative for the treatment of tendinous mallet finger injuries.

## 1. Introduction

Tendinous mallet finger deformities occur when forced flexion to an extended distal interphalangeal (DIP) joint damages the extensor apparatus inserted into the base of the distal phalanx. Although closed trauma is the most common cause of damage, open trauma is also recognized as an etiological cause. Regardless of the type and etiology of the deformity, it is imperative to attempt to repair it, either conservatively or surgically, to reduce the possibility of complications, such as persistent extension lag of the DIP joint and its instability. In addition, mallet finger deformity may cause a swan-neck deformity, as a deformity in one interphalangeal (IP) joint brings about a compensatory deformity in the adjacent IP joint [1,2]. This complex deformity produces pain, functional problems in finger flexion, and the disruption of functional grasp, as well as a dissatisfying cosmetic appearance [3]. Most medical experts advise non-operative treatment, where a finger splint is applied in a DIP extended position with an immobilization period of 6–12 weeks. The most common mean extension lag in the literature is approximately five to ten degrees, but the deficit can be much higher in some patients, leading to permanent functional disability and unpleasing aesthetic results [4,5]. There have been many studies that propose effective treatments for open and chronic mallet injuries, as well as mallet fractures. In open tendon injury cases, meticulous gentle approximation of the ribbon-like flat tendon ends with simple interrupted sutures, and using #4-0 or 5-0 mono filament or braided nylon or Dacron are the most reliable approaches [6]. However, the surgical treatment of acute closed tendinous mallet injuries has not been well established, even though many surgical techniques have been tested to determine the optimal method for treating tendinous mallet finger deformities. In this study, we have proposed a refined technique that uses an open approach and a direct suture method to treat closed tendinous mallet finger injuries.

## 2. Materials and Methods

Between 2019 and 2021, 19 patients with closed mallet fingers without fracture were enrolled in our study; patients with open injuries or bony injuries were excluded from this study. A retrospective chart study was performed on the participants. There were 12 males (63%) and 7 females (37%) aged between 19 and 54 years old (mean: 31 years). The small finger was injured in seven cases (37%), the index finger in six cases (31%), the middle finger in three cases (16%), and the ring finger in three cases (16%). Injury duration was <1 week in eight, <2 weeks in six, 2–4 weeks in three, and 4–8 weeks in two cases. The patients were examined at an outpatient clinic after 6 months from the beginning of treatment, where data on pain, extension lag, and loss of flexion were recorded. The extensor lag before surgery was between −25 and −45 degrees (mean: −29 degrees). Outcomes were classified according to the criteria described by Crawford (Table 1). The Institutional Review Board of Seoul National Bundang University Hospital approved this study (IRB No. B-2301-804-106). This study conformed to the World Medical Association’s Declaration of Helsinki and subsequent amendments and was conducted in accordance with the highest ethical standards.

## 3. Surgical Technique

The procedure was performed under regional anesthesia. After the administration of the local anesthetic, we waited for approximately 10 min for the anesthesia to take effect. A gold post-shaped line was drawn on the dorsal aspect of the distal interphalangeal joint of the affected finger. The incision was made to be wide enough to expose the extensor tendon insertion area and the site of laceration on the base of the distal phalanx. After incision, the dissection plane should be just below the skin dermis and superficial to the extensor paratenon.

We raised the paratenon obliquely to avoid overlapping with the skin incision. Identifying the paratenon is very critical step in this procedure. Sometimes, it is partially torn with the tendon proper, but in most cases, it is well preserved. The retraction of the very thin paratenon edges showed separation of the tendon ends. Both the proximal and distal edges of the extensor were undermined by approximately 3 mm to facilitate the suture. Meticulous gentle approximation of the flat tendon ends using 6-0 PDS (Polydioxanone Suture) was the most reliable approach. Slowly absorbable suture materials, such as Dexon or nonabsorbable nylon, can also be used. The sutures should be placed 1 or 2 mm away from the free ends of the separate tendon with simple interrupted sutures, eliminating the need for multiple passages through these fragile lacerated structures. After tendon repair, the reflected paratenon was reapproximated with 6-0 PDS. Four or five bites with a simple interrupted method were enough. The suture bites one millimeter away from each edge because there is no redundancy in the paratenon tissue. It act as a protective barrier between the skin and tendon, which contribute to reduce friction and prevent tendon adhesion. If paratenon repair is not feasible, like due to the severely torn apart situation, we suggest just performing one or two bites of the nearest edges.

**Figure 1:**
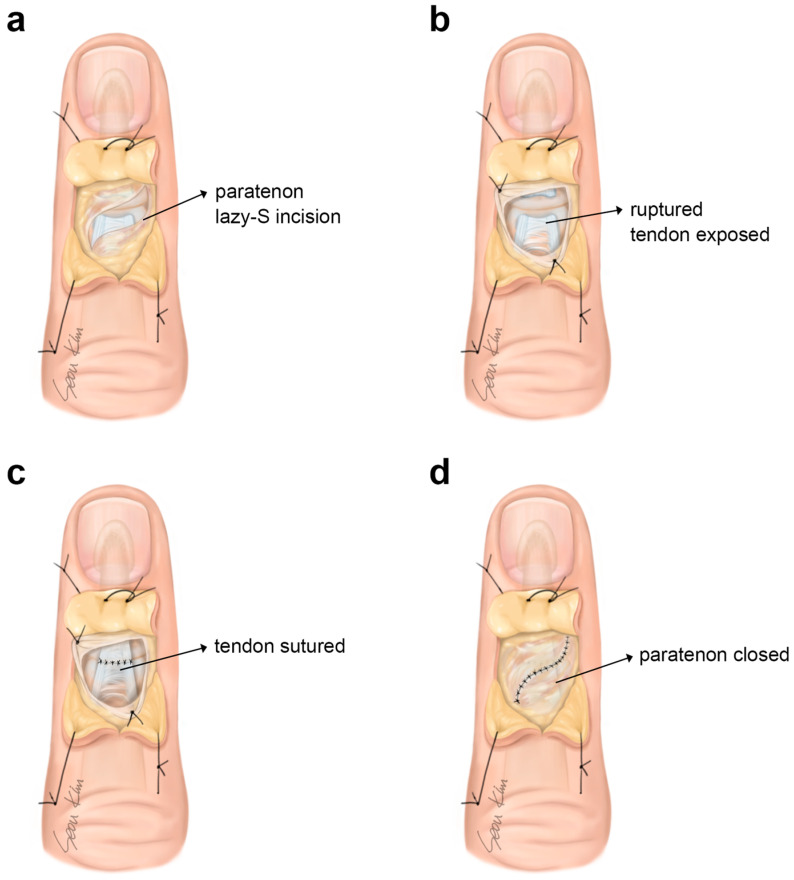
(**a**) After skin incision, the extensor paratenon is designed to be raised obliquely, with no overlap with the skin incision. (**b**) After retraction of the paratenon edges, the ruptured extensor digitorum tendon is identified and undermined. (**c**) The meticulous gentle approximation of the tendon ends is performed with simple interrupted sutures, 1–2 mm away from the free ends of the tendon, to avoid multiple passages through these fragile structures. (**d**) After tendon repair, reflected paratenon is reapproximated to avoid tension adhesion to the skin.

The skin was sutured with one-layer closure using 5-0 nylon thread. Proper immobilization is essential for preservation of tenuous repairs, and delicate care with the application of a fine Kirschner wire to keep the DIP joint in extension ensures the best possible outcome. The DIP joint is fixed in a neutral position or slightly extended using a 0.9 mm Kirschner wire in a retrograde manner through the fingertip across the joint. The pin was cut beneath the skin, and a protective garment or finger orthosis was applied. Postoperatively, only the DIP joint was immobilized in a finger orthosis splint. Patients were encouraged to actively move the proximal interphalangeal and metacarpophalangeal joints, which may enable immediate return-to-work after treatment in some patients. During the orthosis period, the affected finger was maintained on a flat surface in a finger splint to allow the patient and clinician to inspect the skin and cleanse the pin insertion site. The Kirschner wire was removed after 4 weeks, the DIP joint was gradually weaned from immobilization, and night splinting was recommended for two additional weeks (Figure 1).

## 4. Results

The senior author (SC Eun) treated nineteen non-bony mallet finger patients, between 2019 and 2021, using the direct tendon suture and paratenon repair technique. Functional outcomes were evaluated using Crawford’s criteria. The follow-up period was between 3 and 12 months (mean: 5.4 months). All patients resumed their daily activities after 4 weeks. After Kirschner wire removal, the patients started to bend the finger gradually, and it took two or three months to achieve full distal interphalangeal joint motion. The mean initial DIP joint extension lag was 29 degrees (range: 25–45 degrees). The mean DIP joint extension lag at the final follow-up was 6.5 degrees (range: 0–15 degrees; 89% of the patients demonstrated excellent or good outcomes according to Crawford’s evaluation criteria (Table 1). Crawford’s grade was excellent in 3 fingers, good in 14 fingers, and fair in 2 fingers. One patient showed a relapse due to poor compliance with medical advice, but skin necrosis, pin-track/steel-tread infections, and postoperative pain were not observed (Table 2).

**Patient reports**

**Case 1**

**Figure 2:**
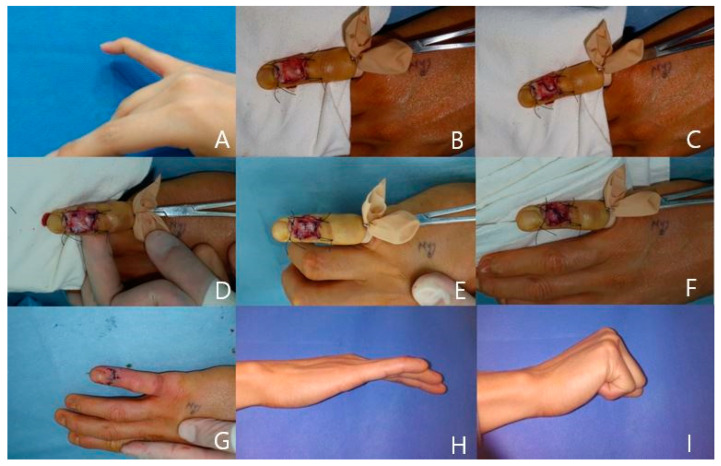
(**A**) A 31-year-old female patient with right little finger mallet injury and extension lag was noted. (**B**) After skin incision and reflection. (**C**) Paratenon incision design. (**D**) Tendon severance noted. (**E**) Tendon sutured. (**F**) Paratenon sutured. The DIP joint is fixed in neutral position by using a 0.9 mm Kirschner wire. (**G**) After skin closure. (**H**,**I**) Finger extension and flexion position after 6 months follow-up period. It shows excellent results.

A 31-year-old woman injured her right fifth finger and was diagnosed with acute tendinous mallet with −55° of extension lag of DIP joint. At 2 weeks after the initial injury, we performed a direct suture with the paratenon repair technique. At 6 months after surgery, the range of motion at the DIP joint improved to 0° of extension with no flexion loss. According to Crawford’s criteria, the patient had an excellent result with no complications (Figure 2).

**Case 2**

**Figure 3:**
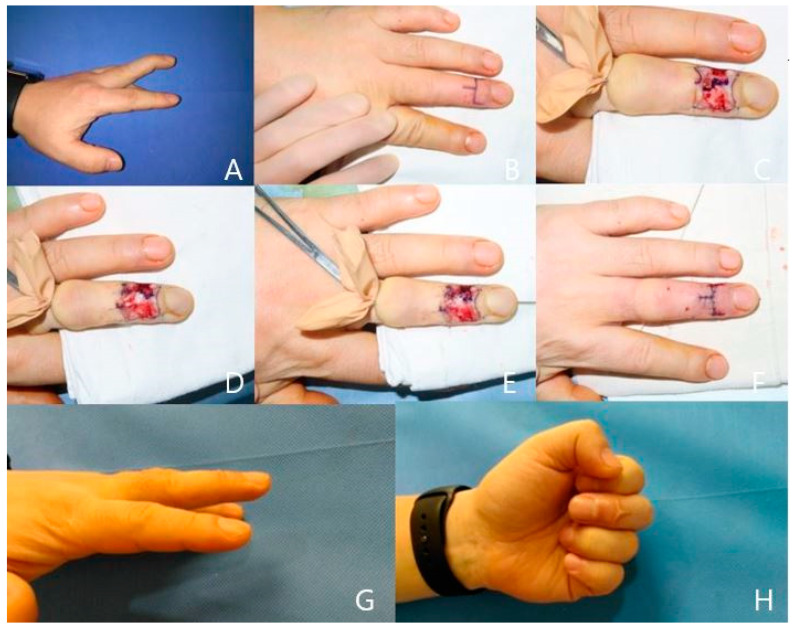
(**A**) A 43-year old male patient with tendinous mallet finger injury on his left third finger. (**B**) The gold-post skin incision. (**C**) Extensor paratenon designed to be raised. (**D**) Ruptured extensor digitorum tendon identified (**E**) Tendon sutured. (**F**) Skin closed. (**G**,**H**) Finger extension and flexion position after a 4-month follow-up period.

A 43-year-old man injured his left third finger and was diagnosed with acute tendinous mallet finger with −25° of extension lag of DIP joint. At 1 weeks after the initial injury, we performed a direct suture with paratenon repair technique. At 4 months after surgery, the range of motion at the DIP joint improved to 0° of extension. According to Crawford’s criteria, the patient had an excellent result (Figure 3).

**Case 3**

**Figure 4:**
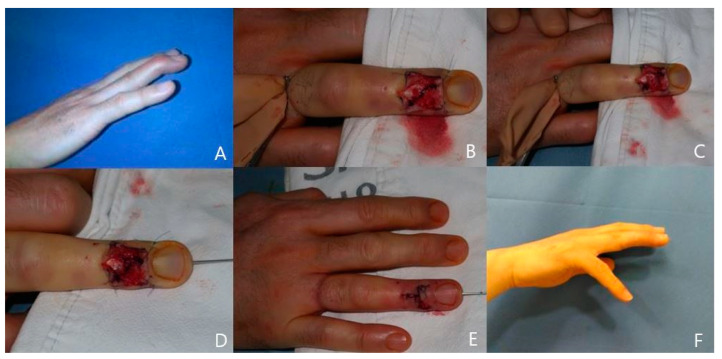
(**A**) A 23-year-old male patient with right fourth finger mallet injury. (**B**) The extensor paratenon incision design. (**C**) Ruptured extensor digitorum tendon exposed. (**D**) Tendon sutured. (**E**) Skin closed. (**F**) Twelve-month postoperative view with no extension lag.

A 23-year-old man injured his right fourth finger and was diagnosed with acute tendinous mallet finger with −35° of extension lag of DIP joint. At 1 weeks after the initial injury, we performed a direct suture with the paratenon repair technique. At 12 months after surgery, range of motion at the DIP joint improved to −5° of extension with no flexion loss. According to Crawford’s criteria, the patient had a good result (Figure 4).

## 5. Discussion

Closed mallet finger is one of the most common chief complaints in the cases of hand trauma, leading to loss of active extension of the DIP joint. Sudden flexion of the DIP joint causes the extensor tendon to be stretched, partially torn, ruptured, or avulsed with a bony fragment at the base of the distal phalanx. The numerous conservative and operative methods described for the treatment of mallet finger deformities indicate that none of these approaches result in good or very good functional outcomes. In particular, acute closed tendinous mallet finger has not been well documented for its gold standard management. Several studies have demonstrated that a very good outcome is possible through continuous splinting of the DIP joint in neutral extension or slight hyperextension for 6–8 weeks in an acute tendinous mallet finger case [6]. However, a high level of patient compliance is an essential prerequisite for successful treatment because the balance of the extensor mechanism tends to be easily disturbed by even a small degree of carelessness. Skin sloughing or nail deformity is more likely to occur in surgical treatment than in conservative treatment. These methods commonly show a greater loss of DIP joint motion including flexion after removal [7,8] because these methods commonly induce scarred healing of the severed tendon, not primary healing, which is an intrinsic limitation.

Several surgical techniques have been introduced to avoid surgical complications; however, some disadvantages still exist. The pull-out wire technique, one of the most widely used conventional treatments, can cause pressure sores or sensory neuroma under the button, prolonging the rehabilitation period [9]. Transarticular K-wire fixation of the DIP joint results in PIP joint flexion contracture and stiffness in some patients. The only apparent advantage of pin fixation compared to orthosis is the provision of a reliable form of immobilization that does not demand much compliance from the patient or need an immediate external orthotic treatment [10]. In addition, the Fowler central slip release technique [11,12], hemilateral band technique [13], percutaneous tenodermodesis [14,15], etc., have a risk of PIP hyperextension, complete detachment of the hemilateral band from the extensor hood or boutonniere deformity, and suture failure following skin breakage [16,17].

However, the technique described in this study, a direct open suture technique, provided excellent and good results in 89% of patients without complication. Our direct suture technique has the following advantages: (1) it allows early postoperative mobilization and leads to early free hand movement, (2) a successful functional recovery is expected, (3) it helps with the healing of the damaged extensor and the normal alignment of fingers is maintained, and (4) it is simple and easy to follow. A mallet finger treatment outcome assessment classification was proposed by Crawford. It is the most commonly used classification for outcome assessment after mallet finger. An excellent outcome is no pain with a full range of motion at the DIP joint, a less than 10-degree extension deficit is a good outcome, a 10–25-degree extension deficit with no pain is a fair outcome, and a more than 25-degree extension deficit or persistent pain is considered a poor outcome [18].

The intrasynovial tendon surface is covered with a lining of cells from the visceral sheet of the synovial sheath, the epitenon, which permits smooth, low-friction tendon gliding under the pulley. The extrasynovial tendon surface is covered with a loose connective tissue called the paratenon [19]. Unlike the epitenon, the paratenon is a loose areolar tissue found on the surface of tendons that has an abundance of vascular networks and has been used to cover the exposed tendon or bone. The paratenon functions as an elastic sleeve that permits some movement of the tendon against the surrounding tissue [20]. The paratenon around the tendon enable good gliding and prevent the adhesion of surrounding tissues. But, when this system is disrupted by injury, fibrosis and adhesions are the common result [21]. Thus, after tendon repair, the paratenon may prevent adhesion by forming a gliding surface for the tendon [22].

In all of our cases, Kirschner wire fixation and external splinting were applied to prevent the flexion of the distal phalanx. However, maintaining the fingers in an extended position for a considerable period may lead to a stiff joint. We removed the K-wire 4 weeks after surgery, which was a bit earlier than for simple wire fixation method. It enables the patient to start early exercise and could achieve better results. After Kirschner wire removal, the patients start to exercise gradual passive and active bending of the finger, and it took two or three months to achieve full distal interphalangeal joint motion.

A major limitation of this study is the small sample size. This is because of the rarity of patients with closed tendinous mallet finger injury. Another limitation is the lack of comparison between the paratenon repair and non-repair groups or other surgical methods. Further studies are required to compare clinical results and the impact of paratenon repair.

In conclusion, this paper describes a series of cases in which the loss of extension of the dip joint caused by a hand injury was successfully treated.

## Figures and Tables

**Table 1:** Crawford’s Criteria Assessment of Mallet Finger.

Grade	Characteristics of DIP Joint
Excellent	Full extension, full flexion, no pain
Good	Extension deficit 0 to 10, gull flexion, no pain
Fair	Extension deficit 10 to 25, any flexion loss, no pain
Poor	Extension deficit > 25, persistent pain

DIP, distal interphalangeal.

**Table 2:** Patient demographics and perioperative evaluations.

No.	Sex	Age (y)	Location	Follow-Up (mo)	Extension Lag (before)	Extension Lag (after)	Crawford’s Evaluation Criteria
1	M	43	Rt 5th F	6	−25	−5	Good
2	F	25	Rt 2nd F	3	−30	−10	Good
3	F	19	Lt 5th F	4	−25	−5	Good
4	M	43	Lt 3rd F	4	−25	0	Excellent
5	M	32	Lt 4th F	3	−35	−5	Good
6	M	23	Lt 2nd F	6	−25	−10	Good
7	M	46	Lt 5th F	9	−30	−5	Good
8	M	45	Lt 5th F	8	−40	−10	Good
9	M	54	Lt 2nd F	4	−35	−25	Poor
10	F	31	Rt 5th F	6	−35	0	Excellent
11	M	25	Lt 2nd F	3	−30	−5	Good
12	M	18	Rt 3rd F	4	−45	−15	Fair
13	M	23	Rt 4th F	12	−35	−5	Good
14	M	35	Lt 2nd F	6	−25	−5	Good
15	M	32	Rt 5th F	4	−30	−5	Good
16	F	61	Rt 4th F	4	−30	−10	Good
17	F	23	Lt 4th F	6	−35	−5	Good
18	F	52	Lt 3rd F	6	−35	−5	Good
19	M	21	Lt 5th F	5	−25	0	Excellent
Mean		31.2	Rt 2nd F	5.4	−29	−6.5	

## Data Availability

The data presented in this study are available on request from the corresponding author.
